# Design of a Training Model for Remote Management of Patients Hospitalized at Home

**DOI:** 10.1007/s40846-020-00553-4

**Published:** 2020-07-09

**Authors:** Patricia Abril-Jiménez, Beatriz Merino-Barbancho, Ivana Lombroni, Samanta Villanueva-Mascato, Irene Mallo, Cecilia Vera-Muñoz, María Teresa Arredondo, Giuseppe Fico

**Affiliations:** Life Supporting Technologies, Universidad Politécnica de Madrid, Madrid, Spain

**Keywords:** Hospitalization at Home, Professionals training, ICT

## Abstract

**Purpose:**

Hospitalization at Home (HaH) has proven to be more efficient and effective than conventional one, but it also requires a higher number of resources and specialised personnel. Information technologies can make this process scalable and allow physicians and nurses to deliver remote healthcare services for patients hospitalized at home. However, a correct and satisfactory usage of technology requires an adequate training of professionals and patients. This paper describes a new model for training healthcare professionals on managing remote ICT-based services for Hospitalization at Home.

**Methods:**

The model was defined based on mix-method that combined the PICO model and a User Centred Design methodology, oriented to identify and discover the healthcare professionals needs and the training instruments in the literature that directly involved these professionals. These aspects were used in the definition and development of the assessment framework of the proposed training model.

**Results:**

A training model for healthcare professionals focused on achieving an effective uptake of complex digital interventions such as Hospitalization at Home was defined. The selected mix-method led to the identification of four different blocks, that were considered as the main areas to include in a training programme. The model identifies measurable elements for assessing acceptability, workability increment and integration into daily clinical practice outcomes, as well as for evaluating the proposed training content and its outcomes.

**Conclusions:**

The proposed training model highlights the key aspects of training health professionals to favour an effective and successful implementation of complex technological healthcare interventions in the context of ICT-based HaH ICT.

## Introduction

Hospitalization at Home (HaH) has proven to be more efficient and effective [1] than conventional hospitalization [2]. It also saves costs and increases the patients’ satisfaction [3]. However, its implementation requires a specialized and qualified clinical team fully dedicated to deliver this kind of service, and this often results in shortages of personnel when the service needs to be scaled to a large population. The recent Covid-19 crisis is leading to a growing demand for these services, and the adoption of remote monitoring technologies to support HaH could help to attend a greater number of patients and to reduce healthcare costs [4].

Nevertheless, the introduction of a new technological process requires to overcome different challenges, such as dealing with change management, engagement, and capacity building aspects, but also providing an adequate training for both, professionals and patients. In particular, the training procedure for a correct introduction and adoption of novel technologies should be accurate and effective, especially when the new technological processes modify the already implemented medical interventions. A right training would allow the healthcare professionals to embrace the required change and to recognise their need to acquire training skills before adopting new technologies. Eventually, it also would help them to teach patients to make informed and secure choices about the proposed treatments or interventions [5].

Previous works have shown that the number of medical professionals willing to adopt changes in the daily routine is rather small [6]. Their most common reactions to this include doubts, rejection, and distrust. Resistance to change is very common in medical institutions, which are very reluctant to make any modification to the established protocols [7].

Remote HaH requires a theoretical and pragmatic knowledge of monitoring devices, technological procedures, and medical protocols, but also a reorganization of daily clinical practices and the integration of the professionals’ behaviours and perceptions in the care processes. In addition, several authors have proposed the need to implement training programs that assess the knowledge in real practice in terms of skills acquisition and cost-effectiveness [8, 9]. However, workability, integration in the real setting environment, satisfaction, workload burden and learning curve are important parameters to properly assess the effectiveness of new ICT-based interventions [10, 11].

This paper describes a model for training healthcare professionals in delivering remote ICT-based HaH services, carried out in the context of the Better@Home project, funded by EIT Health that is supported by EIT, a body from the European Union. The model enables the personalization of the training by considering three aspects: the adaption to the local needs; the identification of the barriers for real setting implementation and the introduction of corrective actions; and the support of the activities and resources reorganization.

## Methodology

The Better@Home project aims to deliver an integrated solution that uses technology to improve the care of patients hospitalised at home. Patients are monitored using devices that measure their vital signs and report back to a healthcare professional, who follows up with the patients remotely. The proposed solution is particularly designed for patients suffering chronic obstructive pulmonary disease, pneumonia, heart failure, and infections.

The system measures different parameters depending on the specific patient and his/her disease (typically oxygen saturation, temperature, respiratory rate, blood pressure, glycaemia, heart frequency or diuresis). These measurements are collected by a set of sensors integrated with a tablet that acts both as a gateway and as the user interface for the training support system. The system generates automatic alerts that need to be integrated with the existing hospital care workflows and protocols (e.g. with the support centre to carry in charge of the administrative tasks). This is essential to allow specialized healthcare professionals focusing on the clinical and medical activities and avoiding workload burden.

**Fig. 1:**
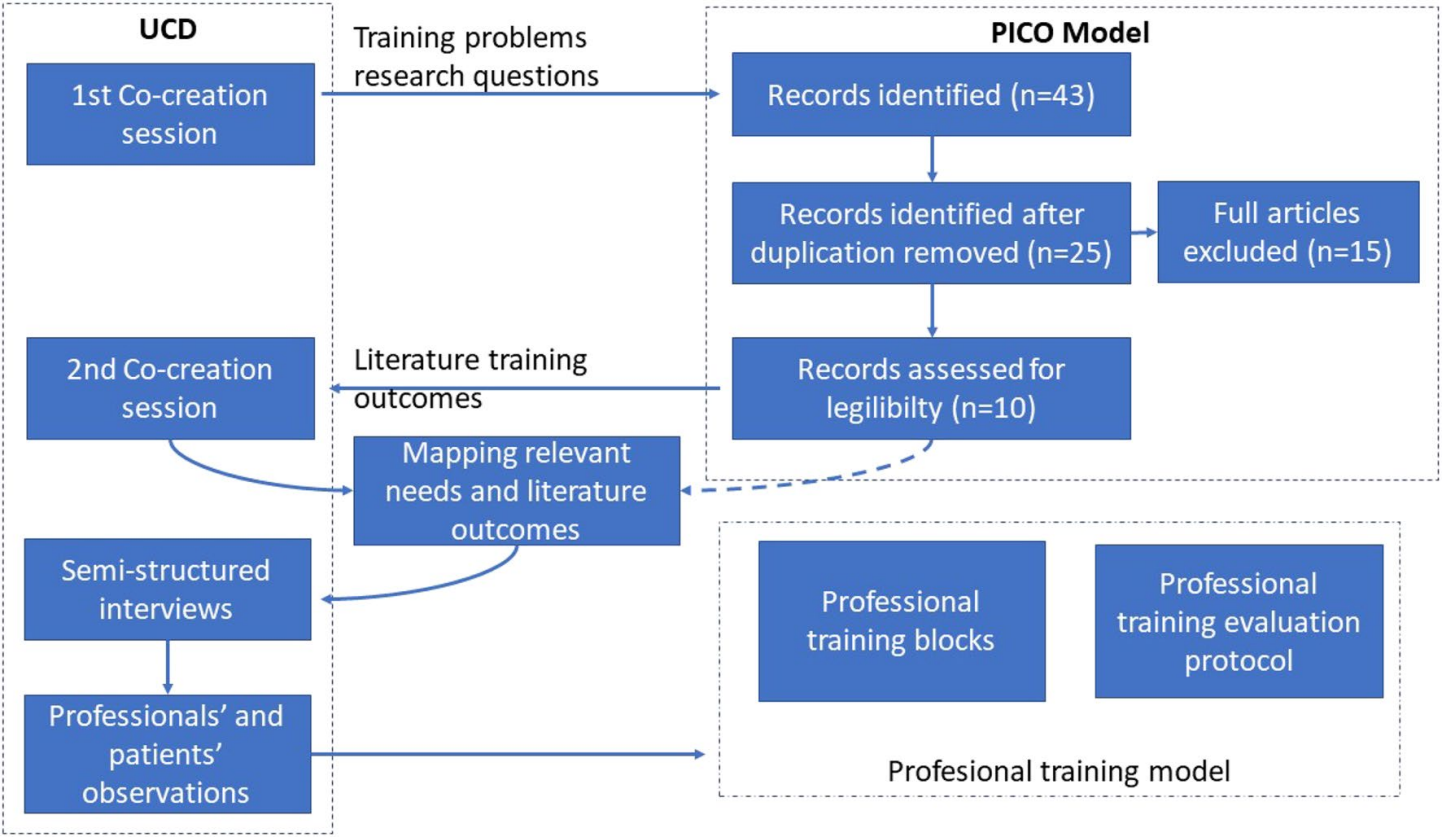
Proposed methodology description

The effective uptake of the proposed solution depends on the successful training of the involved healthcare professionals. In order to structure this training and evaluate its outcomes, we proposed to use a mixed-method based on the PICO (Patient or Population of interest, Intervention, Control or comparation and Outcome of interest) model [12] and a User Center Design (UCD) methodology [13]. The PICO model was used to structure the clinical research questions in connection with evidence syntheses during the literature review, and to discover the right instruments for the training model. Besides, the UCD allowed the inclusion of professional users’ needs and perspectives into the model. The graphical representation of the followed methodology is shown in Fig. 1.

### Analysing the Problem and Planning the Information Gathering and Assessing

The PICO model is a technique used to formulate clinical questions and find the corresponding evidence in the literature. In our case, we utilised it to formulate the research questions that helped us to find the training needs of clinicians regarding the use of new ICT systems applied to HaH (Table 1).

**Table 1:** Research questions co-created with clinicians

Research questions	
RQ1	Is it possible to train the healthcare professional in the inclusion of new technologies in hospital processes and overcome resistance to change and therefore obtain good acceptability to reduce the burden?
RQ2	Is it possible to continue offering patient security and satisfaction in remote care?
RQ3	Is it possible to reduce costs to the Healthcare System by establishing hospitalization at home vs. conventional hospitalization?

The initial research questions were agreed in a co-creation session performed in the Infanta Leonor Hospital (ILH) in Madrid, the pilot site in the Better@Home project, in January 2020. The session involved a total number of ten participants including usability experts, ICT developers, and clinicians with experience on HaH. In this respect, we assured that the users’ feedback was collected and incorporated in the evidence-based research.

Once the research questions were defined, we formulated them in terms of the PICO model, identifying the population (i.e. professionals related to HaH), the considered intervention (i.e. training on new technological skills), and the comparison between different solutions and the outcomes (i.e. elements to evaluate the successfulness of training program). The final questions (Table 2) also described the main barriers for integrating a new technology into an emergency department workflow, including aspects such as trust on the developed technology and patients’ safety.

**Table 2:** Pico model

Questions	Objective
What population are we interested in?	Active professionals directly involved in the interventions and treatments proposed in Better@Home. No restriction about the work type
What kind of interventions are we interested in?	Any kind of questionnaires or evaluation frameworks that cover the main domains and areas of interest (Burnout and overload of the professionals, System general satisfaction, acquired skills, willing to use)
Would the study need a **comparison** group?	Comparison could be with those professionals that do not use any that are not involved in these new practices or training methods
What are the **outcomes** we are interested in?	Improvement documented in the following areas usability perception, workability enhancement, resistance to change

Afterwards, the search terms that best described each of the characteristics of the defined questions were determined (Table 3). These terms were combined using Boolean OR and AND operators to perform an electronic Boolean Search in the following relevant databases: Cumulative Index for Nursing and Allied Health Literature (CINAHL), MEDLINE, PubMed, and Embase. The search comprised all peer-reviewed literature, including non-English citations.

**Table 3:** Terms used for the literature search

Used terms (key words)
Usability
Acceptability
Education material
Health professional
Resistance to change
Skill acquisition
Burnout
Training
Willing to use

The inclusion criteria of the search included full articles that contained: (1) self or interviewer administered questionnaires that measured training outcomes, reporting validity results in the Spanish context; (2) validated questionnaires for emergency services professionals; (3) tested questionnaires, both using their original format or adapted versions.

After completing the search and discarding the duplicated results, two independent researchers made the initial screening of the resulting publications based on a review of their titles and abstracts. This was utilized to select the most promising articles, to categorize the proposed best practices and questionnaires, and to discard those papers that did not exactly fit the defined inclusion criteria.

The resulting list of selected research studies was used to perform a second co-creation session with five healthcare professionals (doctors and nurses) from the ILH in Madrid. This session was conducted using semi-structured interviews and aimed at identifying the professionals’ relevant needs, processes, and other elements that needed to be included in the training, and mapping them with the results obtained in our initial list of research studies after the search. The final objective was to identify the best suitable learning procedure for each of the identified requirements.

The session allowed us to perform a cross-dimensional examination of the HaH intervention protocols uptake among the involved professionals, and their training needs and barriers for adapting the working processes to the current protocols, including the evaluation of elements that hinder the HaH solutions acceptance. The analysis of these interviews provided the indicators to select the training approach that better fitted the professionals’ needs.

### Planning the Training Program

Once all the elements needed for training the healthcare HaH professionals were identified (i.e. common barriers, needs, training blocks, and outcomes to be assessed), the training plan and strategy was defined.

The first step was to perform an observation of the HaH service everyday activities, in order to collect the actual real workflow and adapt the training content to the daily needs. These observations were done in February 2020 in the emergency department of the ILH of Madrid, with the participation 8 healthcare professionals working in the HaH unit (i.e. doctors, nurses and nursing assistants) and 15 patients that were admitted at that moment. The observations collected information both from the clinicians’ and patients’ perspectives. This allowed the detection of elements and key aspects towards the creation of routines and protocols, and the identification of facilitators that would enable integration of the new HaH interventions (including both the technological solutions and the new associated medical protocols) into the professionals' daily routines.

As a result, the evaluation protocol for the training program was defined in terms of hypothesis, outcomes to measure, and instruments to perform the assessment.

## Results

### Barriers Identification and Evaluation Framework

The first literature search resulted in 25 papers retrieved for data extraction. More than half of the papers retrieved were excluded (n = 15), mainly due inappropriate criteria and lack of information on reliability (n = 5) and validity in the Spanish context (n = 10).

The resulted list of research studies (n = 10) provided the validated instruments for measuring the effectiveness, the success of the intervention, the workability increment, and the integration into daily clinical practice outcomes, of using different working environment change management methodologies and training programs for healthcare professionals.

The main key success criterion identified for assessing the training was the normalization of the task, defined as the process that evaluates what people do to make a complex intervention workable and to integrate it into practice [14]. This criterion served during the two co-creation sessions with healthcare professionals to identify a set of barriers to overcome with the training process (i.e. resistance to change and lack of protocol understanding; poor patient-clinician, clinician-clinician and clinician-environmental relationships; and overload).

#### Definition of the Training Blocks

After defining the criteria, the instruments, and the outcomes to be measured, the different elements to be included as part of the training were defined during the two co-creation sessions, using UCD methods. These included the areas, contents, skills, and routines, according to the different needs of the healthcare professionals.

The identified elements were grouped into four different dimensions of training or “training blocks”: (1) Patient HaH context interaction; (2) Confidence and trust; (3) Workability; and (4) Internal organizational context. Each of them addressed specific professionals’ needs to be included as part of the training in order to ensure the successful deployment of a new digital-based complex intervention, and introduced the needed elements to continuously follow up on the outcomes of the training.

#### Patient HaH Context Interaction

This training block instructs and evaluates the immediate social context in which HaH is delivered. The effectiveness of HaH, as a complex health intervention, depends on assumptions that are made regarding the context where it is implemented. In the case of a HaH, the patients acquire an active role that impacts on the effectiveness of the solution, more than when using traditional hospitalization. Besides, the technological environment facilitates both emerging barriers and facilitators.

Understanding the patients’ context is useful to support healthcare professionals in their decision regarding modifications in their treatments or interventions, to consider or emphasize some relevant aspects of the patients’ context (i.e. the caregiver role), or even to stop the intervention in case its effectiveness is not the expected one. In this case, cultural, language, socioeconomic needs, and lack of health literacy are the main barriers to face when selecting a candidate for a HaH service.

This training block also addresses the necessary elements, content, and material to be included as part of the healthcare professional training, so that he/she acquires the necessary skills, support, and knowledge to educate patients in HaH solution understanding. Additional skills, such as an effective communication, the capacity to adapt language style, or to provide attention to the context and the physical comfort, are also necessary to facilitate the patients’ understanding of the HaH context. In this sense, this training block provides healthcare professionals with the tools to detect the patients’ context in advance, so that they can anticipate the barriers for the success of the intervention (i.e. language, cultural, low incomes that make HaH unaffordable, etc.), highlight the available resources that contribute to overcome them, and foresee the intervention success.

Finally, the “Patient HaH context interaction” training block assesses the HaH intervention expectations. The training is considered a success if the healthcare professionals and patients’ expectations are aligned, and if the healthcare professionals acquire the skills to detect misunderstandings regarding expectations and is able to realign the patients’ position.

The elected qualitative data (i.e. PSIQ [12] and CREAC [15]) analyse the congruence among the healthcare professionals and patients’ expectations, the associated disease significance, and the usefulness available training material for patients.

The results of assessing this training block can lead to the identification of a lack of patients understanding of the HaH process and conditions, related to an insufficient acquisition of skills and competences in the new HaH tasks by the healthcare professionals.

#### Confidence and Trust

HaH is a disruptive treatment that changes the patients’ traditional role [2]. The success of this type of complex interventions relies mainly on the patients’ confidence in the healthcare professionals’ work. However, the use of technology in HaH services adds a new aspect to consider in this “Confidence and trust” training block. The theoretical knowledge of the solution may not be enough to transmit trust and confidence to the patients; an understanding of the complex social context of the target population is also required. This training and evaluation block provides healthcare professionals with the methods to help their patients understanding their actions and the processes to be followed during the intervention as part of HaH, including the use of technological devices that support the remote monitoring. The confidence and trust on a technological solution, such as the ones used in the HaH, cannot be considered homogeneous among all the patients. Concerns on privacy aspects have been found to be greater among patients with lower socioeconomic status or patients that lived in rented accommodations.

The training framework needs to measure how comfortable healthcare professionals feel with their level of knowledge about the new HaH protocols after the training. This includes both the medical protocols and the technological processes involved in the intervention. It is expected that healthcare professionals that better acquire and incorporate the new learned tasks into their daily routines will be able to transmit more confidence and trust to their patients [16].

The selected qualitative data (i.e. collected with PSSUQ [17] and CREAC [15] indicate the healthcare professionals’ accountability and confidence. In this sense, the collected data will also allow to identify the role and the weight of those type of activities that contribute to the overall workload (e.g. logistic, family support, etc.). A strong commitment of the healthcare professionals with the complex intervention success requires, not only having the knowledge (accountability), but also the skills to help the patients to understand the solution and trust the caring team. This training block will eventually allow quantifying the patients’ lack of understanding about their condition.

#### Workability

The success of a training program for healthcare professionals should deal with many endogenous factors that can put at risk the success of the intervention delivery. The resistance to change, the burn-out burden among involved professionals, as well as other problems, such as the lack of internal hospital teams’ communication, are identified as the main barriers to an effective professional training. This training block addresses the necessary elements, content, and material to train the healthcare professionals on the daily management of the new intervention processes and protocols.

This training is facilitated when there is a well-defined role specification (i.e. doctor, nurses, nursing assistant), with clear description of the tasks associated to each role. Also, identifying the expected collaboration outline, and proposing communication tools and instruments can be enablers that facilitate the training and the effective integration of new tasks in the daily activities.

Through this training block, we can assess if the training material can regulate the required protocol tasks division based on different professional categories (i.e. clinicians, nurses, administrative, and between different involved teams). This regulation is necessary to allow the different professional actors involved in the intervention to have a clear understanding of their specific tasks, and, to determine if these tasks are aligned with their professional role.

The collected qualitative and quantitative data (i.e. collected with MBI-GS [18] and MPU [19]) measure burn-out and resistance to change due to concerns about job retention and adaptation. This training block allows detecting fears related to the healthcare professionals’ previous experiences and competencies. In this sense, a clear understanding of the involved professionals’ exogenous factors is totally necessary. Besides, measures oriented to the early detection of educational needs related to non-specific skills necessary to collaborate in a HaH unit are required (i.e. collected using PSSUQ and sociodemographic questionnaire [20] and semi-structured interview [21]).

Poor outcomes in this training block allows to identify unresolved questions related to the work allocation and, consequently, on the performance level of the different tasks. Also, it can show different incongruences in the internal team organization.

#### Internal Organization Context

The organizational context is important for the implementation of remote HaH. This training block describes the precise new workflows and processes needed for this implementation, and identifies the links and synergies between the old processes and the new ones.

It includes the workflows management and a cost-effectiveness assessment to validate the new processes and promote the new workflow in the hospital administration. Public university hospitals (e.g. the Infanta Leonor Hospital) should also address additional organizational barriers related to the general training of new healthcare professionals and their integration in daily care processes. In this sense, they must consider formal and official educational objectives, which generally change at a slow pace. This old-new processes mapping is useful to overcome the psychological barriers associated to the integration of the new workflows into the daily tasks.

The collected quantitative and qualitative data in this training block provide information about the impact and cost-effectiveness of implementing new organizational processes (using cost-effectiveness assessment [20] and semi-structured interview results).

## Discussion

In the healthcare domain, where resources are finite or even decreasing in time, any change of resources allocation will likely meet the resistance of the healthcare staff. As a solution, we have proposed a complete training model that allows the identification and assessment of several success outcomes and the definition of the needed training blocks to prepare healthcare professionals in the management of new complex HaH interventions based on remote monitoring technologies.

Our model identifies four different training blocks designed to enable healthcare professional to adapt and work with the new interventions’ protocols in HaH services. In addition, the model provides the elements to validate the effectiveness of deploying complex interventions, not only from a cost-effectiveness perspective but also considering their adoption in the working context by the healthcare professionals.

The model can be adapted according to the specific objectives of a care unit since it incorporates instruments to describe environmental, professional, and management context. Considering the hospital environment and the interactions between the different actors involved in such complex interventions, the model emphasizes the importance of variables such as the congruence between professionals’ and patients’ expectations regarding the new intervention, the confidence in the proposed technology and its ability to demonstrate trust on the proposed treatment, the workability, and the organizational support.

The practical utility of the model lays on the modelling of the professional context, including the relations between professionals as individuals, patients and their context, and the hospital objectives. This provides the required understanding of the healthcare professionals’ specific training conditions, needs, and barriers, and offers need-oriented actions. The model also incorporates a continuous assessment process designed to evaluate each of these actions, as well as to identify deficiencies in the professionals’ training and adapt the training content to tackle them.

Differently from the evaluation theories based on the prediction of outcomes [22, 23], our model identifies the factors and the variables that may affect the adoption and the integration of new processes into the daily routine of the healthcare professionals. Also, in addition of using validated qualitative and quantitative variables for the assessment, the model proposes the use of UCD techniques to ensure the collaboration of the involved healthcare professionals. This way, it provides a better understanding of the real workplace needs and the tools to identify ongoing barriers and shortcomings, and to propose the corrective measures to make the training more effective.

Finally, the model has been designed to be customised and adapted to the requirements of each HaH unit. This is done with mechanisms that analyse the professionals training context and deliver the training blocks according to the needs of the involved professionals. The continuous evaluation framework proposed in the model allows the adaptation of the proposed training structure to the barriers or challenges to be addressed.

## Conclusions

This paper provides a professional training model for remote HaH. We have identified the barriers that difficult the practical implementation of training programs, such as the lack of protocols understanding; a poor patient-clinician, clinician-clinician and clinician-environmental relationships; and the overload in hospitals. To facilitate overcoming these barriers, the proposed model includes the definition of four main training blocks to better identify and map the professionals’ training requirements with the necessary skills to be included in the training programme, and facilitate the integration of these skills and knowledge in their daily working routines. In addition to the requirements, the model identifies variables and assessment instruments to early estimate the outcomes of a training programme. This helps to apply the necessary corrective measures and to maximize the effectiveness of the intervention.

The process for evaluating the healthcare professionals’ training has been created using a robust conceptual model in close collaboration with these professionals. Each of the training blocks includes an evaluation method that assesses the outcomes from a cost-effectiveness perspective and the adoption of new skills by professionals, highlighting its positive contribution to the improvement of the new processes that involve ICT, like the ones focused on hospitalization at home.
